# The Hidden Microbial World in the Gut of the Terrestrial Snail *Cornu aspersum maxima* and the Unexpected Negative Effects of Synbiotics

**DOI:** 10.3390/microorganisms13092127

**Published:** 2025-09-11

**Authors:** Efstratios Efstratiou, Maria V. Alvanou, Dimitrios Loukovitis, Ioannis A. Giantsis, Alexandra Staikou

**Affiliations:** 1 Department of Zoology, School of Biology, Aristotle University of Thessaloniki, 541 24 Thessaloniki, Greece; eefstrab@bio.auth.gr; 2 Department of Animal Science, School of Agriculture, Aristotle University of Thessaloniki, 541 24 Thessaloniki, Greece; malvano@auth.gr; 3 Department of Fisheries and Aquaculture, School of Agricultural Sciences, University of Patras, 302 00 Mesolonghi, Greece; dloukovi@upatras.gr

**Keywords:** *Lactobacillus* *plantarum*, inulin, 16S rRNA sequencing, probiotics, prebiotics

## Abstract

The intestinal microbiome plays a pivotal role in the physiology and health of terrestrial gastropods yet remains largely unexplored. This study aimed to characterize the gut microbial communities of the farmed snail *Cornu aspersum maxima* and to assess the effects of dietary supplementation with the probiotic *Lactobacillus plantarum*, the prebiotic inulin, and their combination (synbiotic) on microbial diversity, snail growth, and survival. In total, 300 sexually immature snails (~9.8 g average body mass, ~5 months old) were assigned to four dietary groups (Control, Probiotic, Prebiotic, Synbiotic), each comprising three replicates of 25 snails. Individuals of similar size were placed in each container within the treatment groups. The Probiotic group received 1.25 mg of *L. plantarum* (1010 CFU/g) per 5 g of feed, the Prebiotic group received 1 g of inulin per 5 g of feed, and the Synbiotic group received both supplements at the same dosages. Over the 60-day trial, the gut microbiota was analyzed via 16S rRNA amplicon sequencing using Oxford Nanopore technology. The results revealed significant treatment-dependent shifts (*p* < 0.05) in microbial composition at both the phylum and genus levels. The dominant bacterial phyla identified were Proteobacteria and Actinobacteria, while a notable increase in unclassified microbial taxa was observed, especially in the inulin-supplemented groups. Despite its known probiotic properties, *L. plantarum* was not detected post-administration, suggesting a transient effect. The synbiotic group exhibited significantly higher microbial diversity (Shannon index, *p* < 0.05) but also the highest mortality rate. All groups showed limited weight gain, with reductions observed after day 30. Potentially pathogenic genera (e.g., *Klebsiella*, *Mycoplasma*, *Staphylococcus*) were detected but showed reduced abundance in the probiotic- and prebiotic-treated groups, suggesting a protective effect. Overall, probiotic supplementation with *L. plantarum* enhanced the abundance of beneficial Actinobacteria and reduced potentially pathogenic taxa, while the prebiotic inulin promoted the growth of unclassified but potentially beneficial genera. However, synbiotic administration, despite increasing microbial diversity, was associated with adverse outcomes including 100% mortality at day 60. These findings highlight both the potential and the risks of dietary manipulation of the snail microbiome, underscoring the need for cautious application of synbiotics in snail farming. They also underscore the dynamic nature of the snail gut microbiome and its responsiveness to dietary interventions, providing valuable insights for sustainable snail farming and future probiotic applications in invertebrate species.

## Simple Summary

The intestinal microbiome is essential to the physiology and well-being of terrestrial gastropods, although it remains mostly understudied. Our work investigates the effects of the probiotic *Lactobacillus plantarum*, the prebiotic inulin, and synbiotic supplementation on the gut microbiome, growth, and survival of the farmed snail *Cornu aspersum maxima*. Probiotic and prebiotic treatments altered gut bacteria, increasing beneficial Actinobacteria and reducing potentially harmful taxa. However, *Lactobacillus plantarum* was not detected after administration, indicating only a transient effect. The synbiotic group showed a significant increase in microbial diversity but was also associated with very high mortality by day 60. Weight gain was minimal across all groups. These results highlight that while probiotics and prebiotics can improve gut health, combining them as synbiotics may lead to adverse outcomes, indicating the need for caution in their application in snail farming.

## 1. Introduction

The intestinal microbiome of terrestrial gastropods is a critical and essential factor for their overall well-being, nutritional efficiency, and ecological role. The microorganisms residing in the digestive tract of snails actively engage in the processes of digestion and nutrient absorption, optimizing food utilization and supporting their physiological health [1,2]. Additionally, the microbiome bolsters the organism’s immune defenses, safeguarding it against pathogenic microorganisms and enhancing immune responses [3,4,5]. Moreover, it is increasingly apparent that the gut microbiota may improve the host’s adaptability by providing metabolic benefits or influencing its ecological preferences, thereby contributing to the maintenance of ecosystem equilibrium [6,7].

Furthermore, the decline in wild gastropod populations in their natural environment has become increasingly evident, with the growing trend of intensive harvesting of edible species significantly exacerbating the risk of their extinction [8]. The need for organized farming of edible snails has thus become imperative, with the farming of *Cornu aspersum maxima* proving to be an important, flexible, and sustainable activity offering significant economic, environmental, and biological benefits. From the production of food [9,10] and cosmetic products [11,12] to the recycling of organic waste [13] and the reduction of the environmental footprint of livestock farming [14], snails of this species provide numerous opportunities that promote sustainability. These applications also contribute significantly to economic development [15]. The demand for snail products demonstrates a continuous increase, making their farming applications a promising future sector. Nevertheless, the limited knowledge regarding biology, ethology, and production processes in snail farming has led to significant concerns [15]. Additionally, several challenges arise in the farming process of these animals, as snails are sensitive to climatic changes [16] and infections [17], which causes the management of the rearing conditions to be demanding and may potentially affect both production and the viability of the industry.

In addition to the appropriate infrastructure of the facilities and the required safety conditions, the foundation for the successful farming of terrestrial snails lies in thorough knowledge of the factors affecting growth and reproduction [18,19], as well as adequate and balanced nutrition, which affects critical aspects of their life cycle traits [18,20,21,22]. The diet of snails must be rich in essential nutrients such as calcium, dietary fibers, and vitamins to ensure their optimal well-being [23,24]. The inclusion of probiotics in snail farming can provide significant health benefits by enhancing the gut microbiota, supporting the immune system [25,26], improving shell quality, reducing the risk of infections and other diseases, and potentially promoting their reproductive capacity and growth [27,28]. Moreover, the gut microbiota is also enhanced by the consumption of prebiotics, which, according to the most recent definition, are substrates selectively utilized by host microorganisms that provide health benefits to the living organism [29]. Many different nutrients, such as pectin, cellulose, and xylan, promote the growth of various gut microorganisms. Prebiotics should not undergo extensive metabolism but instead trigger targeted metabolic processes that benefit the health of the host’s ecosystem [30,31]. Overall, the application of probiotics and prebiotics reduces the need for pharmaceutical treatments, leading to a reduction in operational costs and an increase in both the production and quality of invertebrates [32].

Strains of the genus *Lactobacillus* are systematically used as potential probiotics, demonstrating their proven safe consumption [33,34]. Some of these are members of the indigenous microbiota of *Cornu aspersum* (O.F Müller, 1774) snails and are capable of surviving and maintaining active metabolism even during extended periods of dormancy [35]. Furthermore, the presence of these variants plays a significant role in the recovery of snails previously infected by pathogenic strains of *Listeria monocytogenes*, enhancing their natural defense mechanisms and promoting their survival in environmental conditions that favor the development of infections [36].

In recent years, there has been an increase in the use of probiotic-enriched animal feeds in livestock farming, with diverse types of probiotic strains commercially available. These probiotics are widely used in animal husbandry and aquaculture, as they have been shown to enhance growth, feed conversion, and overall animal health [37,38,39,40,41]. Simultaneously, a wide variety of feed additives are utilized in studies investigating the effect of prebiotics on the gastrointestinal microbiota and the general health status of animals. In most cases, studies focus on farmed poultry and pigs, reporting positive effects on average daily growth [42], weight gain, and feed conversion, as well as beneficial impacts on the development of *Bifidobacterium* and *Lactobacillus* bacteria, alongside the inhibition of *Escherichia coli* growth and a reduction in the number of other potentially pathogenic bacteria [43]. The prebiotics mostly used in animal diets include fructo-oligosaccharides (FOSs), galacto-oligosaccharides (GOSs), inulin, isomalto-oligosaccharides (IMOs), xylo-oligosaccharides (XOSs), lactitol, lactulose, and cereal fibers [44,45]. The fermentation of plant fibers and other indigestible components leads to the production of short-chain fatty acids (SCFAs), which lower the pH of the intestinal environment, thereby creating unfavorable conditions for the growth of harmful bacteria [46,47]. As a result, enhancements are observed in growth, fertility, and egg quality, while shell resistance is further strengthened due to optimized absorption of nutrients such as calcium.

The cellular mechanisms underlying the positive impact of probiotics on physiological aspects other than snails’ immune response remain unclear and have not been sufficiently studied. In a recent study, we have focused on the nutritional effects of *Lactobacillus plantarum* and found evidence for beneficial effects on the physiology of terrestrial snails [48].

When developing the composition of prebiotic and probiotic formulations, determining the optimal combination is critical for their effectiveness. In this context, this study aims to evaluate the effects of dietary supplementation with the probiotic strain *L. plantarum*, the prebiotic inulin, and their combination as a synbiotic (*L. plantarum* + inulin) on *Cornu aspersum maxima*. Specifically, it investigates how these additives—individually and in combination—affect two main parameters: (i) the growth performance of the snails, and (ii) the composition and diversity of their intestinal microbiome across different treatment groups.

## 2. Materials and Methods

### 2.1. Snail Rearing and Sample Collection

A total of 300 sexually immature *Cornu aspersum maxima* individuals, with an average body mass of 9.79 ± 0.46 g and an approximate age of ~5 months, were obtained from a snail farm and were distributed into four treatment groups and placed in glass containers (20 × 20 × 40 cm). Each treatment group consisted of three replicates with 25 snails. Individuals of similar size were carefully allocated to each glass container within the groups to ensure uniformity of experimental material. The temperature of the rearing room was adjusted to 22 ± 2 °C, while humidity levels were maintained between 70 and 90% by placing a wet sponge inside the containers. The experiment was conducted under a 14 h light/10 h dark photoperiod using cool white lamps. The snails were acclimatized for one week to the specified conditions without food. Dead snails were replaced with individuals of equivalent size to ensure that the snail density remained consistent across all containers. This replacement strategy ensured that any differences observed in the intestinal microflora between the containers would not be attributed to variations in density. Furthermore, the newly introduced snails were excluded from tissue sampling.

Following the acclimation period, the snails were provided with a commercial ground feed ration (Zoothreptiki Animal Nutrition SA—Sapounas Bros SA, Volos, Greece, cat. number 23A) (Table 1). The feed was divided into four distinct experimental groups. The first group received the basal diet without any supplementation of probiotics or prebiotics. The second and third groups were enriched with 1.25 mg of *L. plantarum* and 1 g of inulin per 5 g of feed, respectively. The fourth group received both 1.25 mg of *L. plantarum* and 1 g of inulin. These specific doses were determined based on previous studies investigating the effects of probiotics and prebiotics in snails and other farmed animals [25,36,49]. In Dushku et al. [25], adult snails weighing approximately 15–20 g were fed 1 g/day of commercial snail food containing 5 × 10^8^ CFU of *L. plantarum* for 10 consecutive days, resulting in enhanced immunomodulatory responses, including increased hemocyte phagocytic activity and nitric oxide production. Similarly, in Dushku et al. [36], snails weighing 15–18 g were fed with food containing 5 × 10^9^ CFU of *L. plantarum* daily after dysbiosis induction, which led to restoration of the gut microbial balance and improved health indicators. The inulin dose was based on the study by Xia et al. [49], where dietary supplementation with 1%, 2%, and 4% inulin was tested in broiler chickens over a 42-day period, and the 2% dose (20 g/kg feed) produced the best effects on gut health and growth performance. Although species-specific physiological differences exist, this study provided a reference framework for determining a functionally relevant inulin dose. The probiotic strain *L. plantarum*, in powder form, was sourced from Swanson company (Fargo, ND, USA, product code: SWV-19016), with each capsule containing 25 mg and 10 billion CFU/g (colony forming units) at the time of manufacture, providing an effective level of bacteria. Inulin powder (product code: SWU520), derived from chicory roots, was also obtained from Swanson.

**Figure 1:**
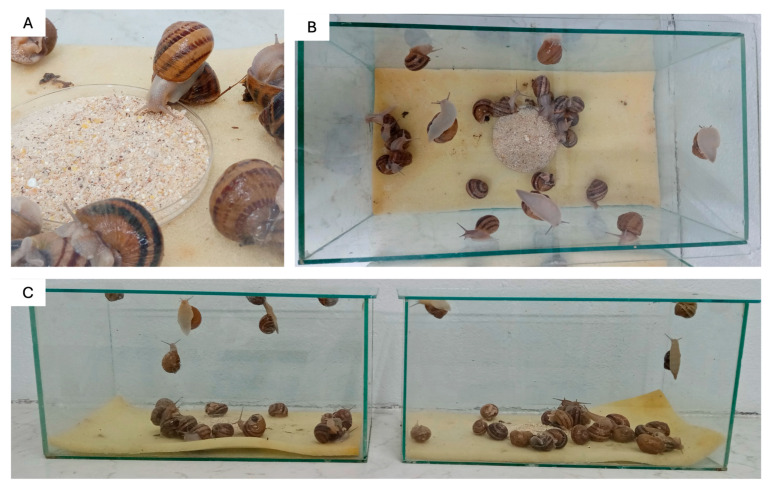
Housing and feeding of experimental snails. (**A**) Snails at close distance while approaching the provided food. (**B**) Top view of the glass container used for maintenance. (**C**) Side view of two containers showing the housing conditions.

Prior to placement in the Petri dishes, each additive (*L. plantarum* and/or inulin) was carefully mixed into the 5 g of ground basal feed using a sterile spatula to ensure homogenous distribution throughout the ration. This mixing process was repeated for each replicate and group, resulting in consistent concentrations of the active substances across all experimental units. The prepared rations were then placed in Petri dishes of uniform size and transferred to the rearing containers. Although the portions provided were equal in quantity (5 g per dish), the goal was not to enforce equal consumption by the snails, but rather, to ensure continuous and equal access to feed for all individuals throughout the trial period (Figure 1). This allowed each snail the opportunity to consume the supplemented feed at least once, fulfilling the experimental objective. No depletion of feed was observed in any group during the study.

Equal portions of the experimental ration were placed in petri dishes of uniform size and subsequently transferred to glass containers. Snails were allowed ad libitum access to food from the beginning until the end of the trial period. The group receiving the base diet without any supplementation served as the Control, while the second, third, and fourth groups were labeled as Probiotic, Prebiotic, and Synbiotic, respectively. The experiment lasted for 60 days (June–August 2024). A total of two samplings were conducted every 30 days. Five days before sampling, snails were deprived of food to ensure that the intestine would be empty during sampling. The previous day before sampling, individuals were drawn out of the glass containers and were immediately frozen at −25 °C. A total of 10 snails were randomly selected from each treatment and were divided into two groups of five individuals and pooled for analysis. For tissue sampling, snails were thawed, and the shell was discarded to reveal the visceral mass. The intestine was then carefully excised, rinsed in distilled water, placed in Eppendorf tubes, and stored at −80 °C for subsequent analyses. In total, eight sample groups were created named “30_CONTROL”, “30_PROBIOTIC”, “30_PREBIOTIC”, and “30_SYNBIOTIC” for the sampling on the 30th day of the experiment and “60_CONTROL”, “60_PROBIOTIC”, “60_PREBIOTIC”, and “60_SYNBIOTIC” for the sampling on the 60th day of the experiment.

### 2.2. Molecular Analysis

Total microbial DNA was isolated from the homogenized gut of *C. aspersum maxima* specimens after thawing, utilizing the Macherey-Nagel™ NucleoSpin™ DNA Stool Kit (Macherey-Nagel, Düren, Germany). After DNA extraction, the concentration and integrity of all DNA samples were assessed using a Q3000 microvolume spectrophotometer (Quawell, San Jose, CA, USA). The extracted DNA was amplified using the modified primer pair 27F (5′-AGRGTTYGATYMTGGCTCAG-3′) and 1492R (5′-CGGYTACCTTGTTACGACTT-3′), targeting the V1-V9 hypervariable region of the 16S rRNA gene (∼1500 bp). PCR amplification of the 16S rRNA amplicons was carried out with the KAPA2G™ Robust HotStart ReadyMix PCR Kit (Kapa Biosystems, Wilmington, MA, USA) in a total volume of 15 μL, containing the primer pair (50 nM each), PCR-grade water, and DNA sample (∼50–100 ng). Amplification conditions included an initial denaturation at 95 °C for 3 min, followed by 30 cycles of 95 °C for 15 s, 55 °C for 15 s, and 72 °C for 30 s, with a final extension at 72 °C for 1 min. The resulting amplicons were verified using agarose gel electrophoresis to confirm successful amplification of the 16S rRNA gene. Finally, the purity and concentration of the PCR products were assessed using a Q3000 UV-spectrometer (Quawell, San Jose, CA, USA).

### 2.3. Library Preparation and Amplicon Sequencing

PCR products were purified using AMPure^®^ XP beads (Beckman Coulter, Brea, CA, USA). The purified 16S rRNA amplicons were prepared for sequencing via ligation with the Native Barcoding Kit 96 V14 (SQK-NBD114.96, Oxford Nanopore Technologies plc., Oxford Science Park, Oxford, UK), where each PCR sample was assigned a unique barcode. The final pooled library, consisting of all barcoded PCR products, was loaded into a flow cell (version R.10.4.1, Oxford Nanopore Technologies plc., Oxford Science Park, Oxford, UK) and inserted into a MinION-Mk1B sequencing device (Oxford Nanopore Technologies plc., Oxford Science Park, Oxford, UK). The sequencing parameters were set using the MinKNOW software v. 24.02.10 (Oxford Nanopore Technologies plc., Oxford Science Park, Oxford, UK).

### 2.4. Bioinformatic and Statistical Analysis

Parameters examined herein (mortality, WGR) were tested for significance at the 5% level (*p* < 0.05) by using one-way (GraphPad Instat 3.0) analysis of variance (ANOVA). Values are presented as means ± S.D. Friedman’s non-parametric test, followed by Dunn’s post-test, was performed to re-analyze and cross-examine our data. Post hoc comparisons were corrected by a Bonferroni test.

Raw sequencing data (POD5 files) were base called using algorithms integrated into Dorado software version 1.1.1 (Oxford Nanopore Technologies plc., Science Park, Oxford, UK), where reads were demultiplexed according to the corresponding barcodes. High purity 16S rRNA sequences were generated by removing barcodes, adapters, and primer sequences. Reads with lengths below 1000 bp or above 2000 bp were excluded, and a quality score (Q-score) threshold of 15 was applied.

For the taxonomic analysis, the QIIME 2 bioinformatics platform (version 2024.5.0) [50] was utilized alongside the SILVA 138 SSU Ref non-redundant (NR) database (available at https://www.arb-silva.de/documentation/release-123, accessed on 10 June 2025, applying a minimum sequence similarity threshold of 90% for classification. Alpha rarefaction curves were generated at the genus level, based on the Shannon diversity index, to investigate the relationship between sequencing depth and bacterial richness across barcoded PCR samples.

For the diversity analyses, sequenced amplicons were categorized according to sample type (CONTROL_30, PROBIOTIC_30, PREBIOTIC_30, SYNBIOTIC_30, CONTROL_60, PROBIOTIC_60, PREBIOTIC_60, SYNBIOTIC_60), resulting in the formation of eight distinct groups.

Beta diversity analysis was conducted using the Bray–Curtis dissimilarity index [51] to evaluate community composition differences among the groups. To facilitate visualization and comparison of taxonomic profiles, several graphical outputs were generated, including taxon bar plots, Emperor plot, heatmap, hierarchical clustering dendrogram, Non-metric Multidimensional Scaling (NMDS) plot, and Venn diagrams, each representing the composition and abundance of bacterial taxa between sample groups. More specifically, microbial community dissimilarities were assessed using a Bray–Curtis distance matrix calculated from OTU abundance data. Distance matrices were computed using the vegdist function from the vegan package in R. Samples were hierarchically clustered based on average linkage (UPGMA) and visualized through a dendrogram. The NMDS plot was performed to represent the community dissimilarities in reduced dimensions, visualized using both base R plotting functions and enhanced with ggplot2 for clarity.

## 3. Results

### 3.1. Growth Performance and Mortality

Table 2 demonstrates the mortality (%) and the weight values of the four groups studied: Control, Probiotic, Prebiotic, and Synbiotic, measured after two time intervals (30 and 60 days). During the rearing period, mortality increased over time in all groups. The Control group exhibited exceptionally low mortality after 30 days (only 5.71%). However, a notable increase was observed after 60 days (68.57%), indicating a progressive rise in mortality in the absence of any dietary intervention. The group administered *L. plantarum* (Probiotic) showed a marginally higher and statistically significant mortality compared to the Control group after both 30 days (14.29% versus 5.71%) and 60 days (51.43% versus 68.57%).

The snails receiving inulin (Prebiotic) also demonstrated higher mortality than the Control group after 30 days (20% versus 5.71%) and 60 days (62.86% versus 68.57%). The difference was statistically significant only for the 30-day measurement. The group receiving the combination of Probiotic and Prebiotic (as Synbiotic) exhibited the highest mortality among all groups, both after 30 days (48.57%) and 60 days (100%), which was statistically significant. While mortality after 30 days was already higher compared to the Control and the other treatment groups, the difference became more pronounced after 60 days, with mortality reaching 100%, almost double that of the Control group.

Taken together, the findings indicate that, although slight differences among the groups were observed, the group fed with the combination of *L. plantarum* and inulin exhibited a greater increase in mortality, with the disparity becoming more evident after 60 days.

After 30 days, an increase in weight was observed across all groups. The Probiotic group showed the lowest statistically significant increase in weight compared to Control, while the other groups exhibited a more substantial weight gain, with the combination of *L. plantarum* and inulin demonstrating the greatest increase. After 60 days, the weight of snails decreased in all groups compared to their weight at 30 days. The Probiotic group experienced the greatest weight reduction, which was the highest among all groups.

Analyzing the weight growth rate (WGR) at 30 days, it is evident that the Control group had the highest value (14.18%), followed by the Prebiotic group (13.89%) and the Synbiotic (13.27%). The Probiotic group displayed the lowest WGR (11.13%). At 60 days, the WGR of all groups showed a notable decline. The Control group still performed the best, with a growth rate of 8.99%, while the Probiotic and Prebiotic groups exhibited much lower WGRs (2.97% and 1.58%, respectively). The *L. plantarum* and inulin (Synbiotic) group continued to demonstrate a favorable outcome (7.76%), although this was also lower compared to the WGR at 30 days. The above weight changes were not statistically significant, probably due to the high SD values, occurring from the uneven growth performance characterizing the individuals within a snail population.

### 3.2. 16S rRNA Amplicon Sequencing

**Figure 2:**
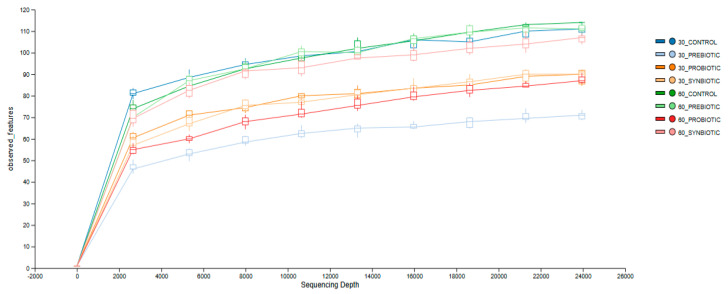
Alpha rarefaction curves of all barcoded 16S rRNA amplicon samples at the genus level using observed features.

After performing basecalling and filtering raw sequencing data, a total of 541,954 reads were obtained. The number of 16S rRNA amplicon reads per barcode ranged from 28,262 to 107,414, with an average of 67,744 reads per sample. To ensure comparability, all samples were rarefied to a uniform depth of 28,262 reads for the calculation of core diversity metrics. Alpha rarefaction analysis, as depicted in Figure 2, shows that the rarefaction curves for bacterial populations at the genus taxonomic level have nearly reached a plateau phase. This indicates that the sequencing depth was adequate and further increases in the number of reads would not significantly alter the number of genera detected. These findings suggest that the sequencing effort was sufficient to capture most of the bacterial diversity at the genus level, and additional sequencing would yield diminishing returns in terms of new genus discoveries.

### 3.3. Microbial Diversity in the Gut of the Land Species Cornu aspersum maxima

#### 3.3.1. Phylum Classification

According to the taxonomic analysis, the gut microbiome is predominantly composed of the bacterial phylum Proteobacteria (formerly Pseudomonadota), with relative abundances ranging from 20.65% to 83.68%. Actinobacteria was also detected in considerable proportions ranging from 3.47% to 56.05%, while a considerable share of the microbial community was attributed to an unclassified phylum ranging from 3.90% to 34.99%, followed by Firmicutes with an abundance rate ranging between groups from 1.56% to 11.31%. Additionally, Bacteroidota was detected at low levels across the dataset, with values ranging from 0.2% to 8.1%. In contrast, other phyla such as Bdellovibrionota, Fusobacteriota, Gemmatimonadota, Verrucomicrobiota, Sumerlaeota, Planctomycetota, Acidobacteriota, and Deinococcota were present at negligible levels, typically exceeding only 1% of relative abundance.

Marked variability was observed between the phyla Actinobacteria and Proteobacteria, suggesting substantial inter-sample heterogeneity. A comparison between the 30_CONTROL and 60_CONTROL groups revealed major fluctuations in the relative abundances of the dominant phyla. Proteobacteria demonstrated a marked increase in abundance (58.42%), Firmicutes were decreased, and Actinobacteria from the main phylum exhibited a substantial decrease (15.58%). The presence of Bacteroidota remained consistently low, without notable differences between the timepoints, while the unassigned microbes exhibited a small increase (13.46%).

In the comparison of the 30_PREBIOTIC and 60_PREBIOTIC groups, significant shifts in microbial composition were observed. Although Proteobacteria remained one of the dominant phyla at day 30 (83.68%), its relative abundance declined substantially by day 60 (51.64%), in contrast to unclassified phyla, which presented a significant increase of 34.99%. Firmicutes showed a slight decrease (1.56%), while Actinobacteria experienced the most prominent change, with a significant reduction in abundance (3.47%). In contrast, Bacteroidota persisted at low abundance, with a minor uptick observed by day 60.

Similarly, in the comparison between the 30_PROBIOTIC and 60_PROBIOTIC groups, a notable increase in Actinobacteria was recorded by day 60 (28.36%), accompanied by a modest decline in Firmicutes (3.80%) and a downward trend in Proteobacteria (43.21%). Bacteroidota remained at low levels across both timepoints, showing no meaningful differences. Taxonomically unresolved phyla showed an upward trend (20.69%) after 60 days.

**Figure 3:**
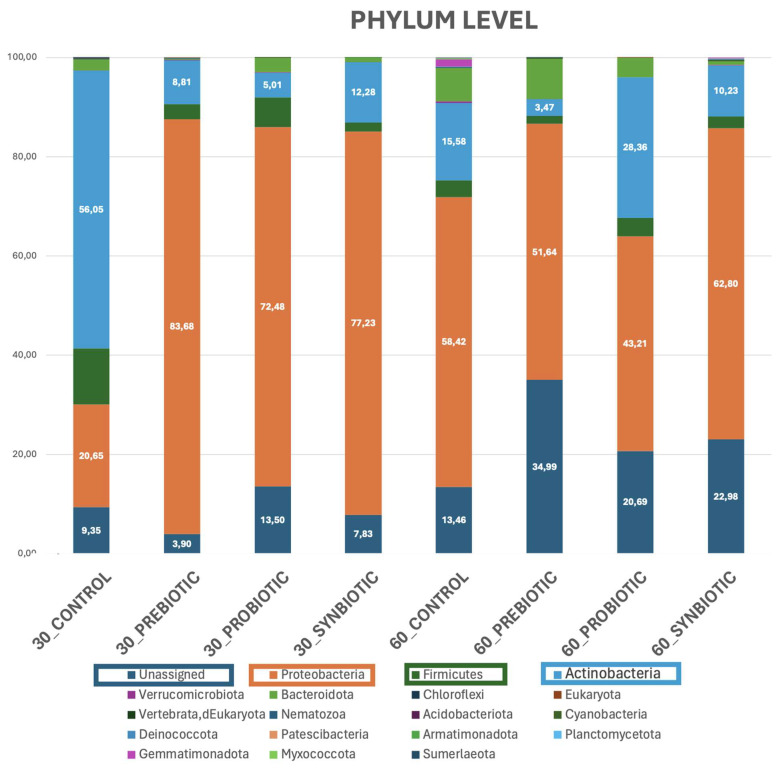
Taxon bar plots of the eight sample groups at the phylum taxonomic level. Numbers in the bar plots represent the percentage of abundance of the phyla Proteobacteria and Actinobacteria as well as the percentage of the unassigned phyla.

Finally, analysis of the 30_SYNBIOTIC and 60_SYNBIOTIC groups revealed that the abundance rate in phyla of unknown classification was tripled (22.98%). Firmicutes maintained an almost constant abundance over time, while Actinobacteria demonstrated a slight downward trend. Moreover, Proteobacteria showed a decreasing pattern (62.80%), and Bacteroidota remained relatively low (ranging from 2.97% to 3.93%) without statistically relevant changes in abundance (Figure 3).

#### 3.3.2. Genus Classification

The comparative analysis of the gut microbiome across all experimental groups revealed distinct variations in bacterial composition at the genus level, reflecting both temporal changes and the effects of different treatments. Genera with a relative abundance below 1% in all groups were classified under the category “others”.

In the control groups, microbiome profiling indicated notable differences between the 30_CONTROL and 60_CONTROL timepoints apart from the “others” group, where the abundance did not show much variation. In the 30_CONTROL group, the bacterial community was dominated by *Cutibacterium* (44.06%), followed by *Lawsonella* (6.40%), *Staphylococcus* (4.60%), *Enterobacter* (3.36%), and *Klebsiella* (3.28%). Genera such as *Streptococcus* (2.92%), *Corynebacterium* (2.25%), and *Lactococcus* (2.19%) were also detected in lower abundances. By day 60, the 60_CONTROL group exhibited substantial shifts, with a remarkable increase in *Buttiauxella* (13.29%) and elevated levels of *Enterobacter* (11.51%), in contrast to *Cutibacterium*, which showed a significant decline (11.41%) from its earlier dominance. Additionally, new genera such as the *Allorhizobium–Neorhizobium–Pararhizobium–Rhizobium* complex (7.30%) and *Sphingobacterium* (6.34%) appeared. Additionally, the genera *Acinetobacter* (2.52%), *Pseudomonas* (2.43%), and *Brevundimonas* (1.99%) were also identified, indicating a more diverse microbial landscape.

The probiotic intervention also resulted in considerable microbial restructuring. In the 30_PROBIOTIC group, *Klebsiella* (36.61%) and *Enterobacter* (27.05%) dominated the bacterial community, accompanied by unclassified genera (13.5%) and “others” (11.85%), *Cutibacterium* (3.89%), and *Staphylococcus* (2.44%). By day 60, the genus *Cutibacterium* had emerged as the most abundant (24.07%), while unclassified microbes remained prominent (20.69%). The abundances of *Enterobacter* (8.62%) and *Klebsiella* (7.33%) showed marked reductions. At the same time, the presence of *Buttiauxella* increased (7.59%), and previously undetected genera such as *Micrococcus* (2.33%), *Acinetobacter* (2.24%), and *Arcticibacter* (1.41%) became more distinguishable, indicating a diversification of the microbial profile.

In the prebiotic groups, analysis of the 30_PREBIOTIC and 60_PREBIOTIC samples revealed both conserved and altered microbial features. On day 30, the microbiota was predominantly composed of *Klebsiella* (43.29%) and *Enterobacter* (34.03%), with a moderate presence of *Cutibacterium* (7.65%). Minor genera such as *Lactococcus* (2.14%) and *Buttiauxella* (1.86%) were also observed. By day 60, although *Klebsiella* (19.80%) and *Enterobacter* (18.36%) remained prevalent, a substantial proportion of the microbiota (34.99%) was assigned to an unclassified genus, marking a distinct shift in microbial composition. Moreover, newly detected genera such as *Dysgonomonas* (4.86%), *Macellibacteroides* (2.87%), and *Shewanella* (2.78%) further highlighted the dynamic nature of the gut ecosystem in response to prebiotic supplementation.

**Figure 4:**
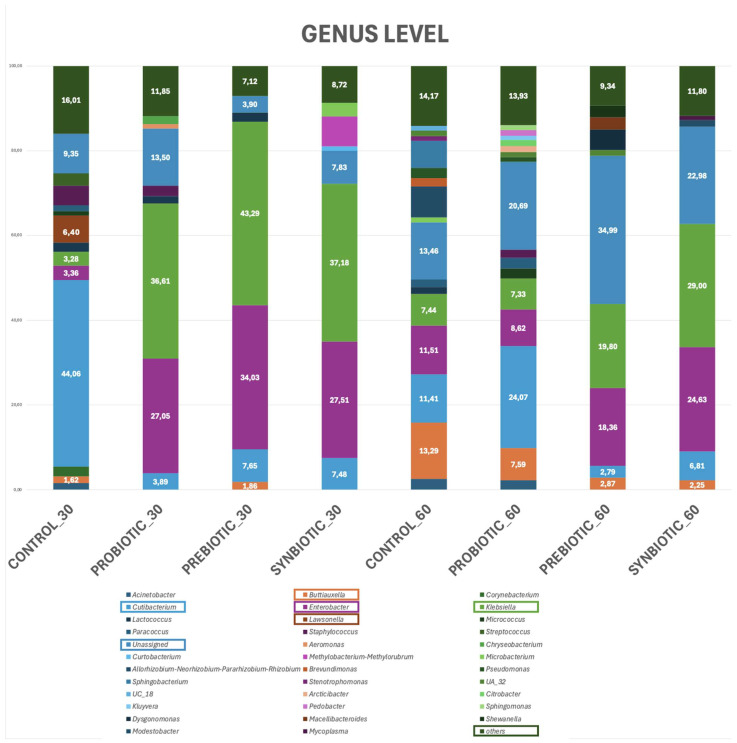
Taxon bar plots of the eight sample groups at the genus taxonomic level. Numbers in the bar plots represent the percentage of abundance of the genera *Curibacterium*, *Buttiauxella*, *Klebsiella*, *Enterobacter*, *Lawsonella*, unassigned, and genera with a relative abundance below 1% (others).

In the synbiotic groups, significant alterations in bacterial composition were also observed over time. By day 30, the microbial community was largely composed of *Klebsiella* (37.18%) and *Enterobacter* (27.51%), with additional notable contributions from *Cutibacterium* (7.48%) and *Methylobacterium–Methylorubrum* (7.07%). Minor genera included *Microbacterium* (3.18%) and *Curtobacterium* (1.02%). By day 60, although *Klebsiella* (29.00%) and *Enterobacter* (24.63%) remained dominant, both exhibited a reduction in relative abundance. In contrast, unclassified genera increased significantly from 7.83% to 22.98%, as did “others”, increasing from 8.72% to 11.80%. Furthermore, new genera such as *Buttiauxella* (2.25%), *Modestobacter* (1.52%), and *Mycoplasma* (1.00%) emerged (Figure 4).

### 3.4. Diversity Analysis

A beta diversity analysis was conducted using the Bray–Curtis dissimilarity index to assess the variations in genus composition across all possible pairwise sample groups, based on bacterial counts within each group. In Table 3, the structure of the experimental design is depicted, combining both treatment type and barcodes. Each treatment group (Control, Probiotic, Prebiotic, Synbiotic) is represented at two timepoints: day 30 and day 60. For example, BC01 and BC09 are control samples collected after 30 and 60 days, respectively. This structure enables the investigation of how both the type of dietary intervention and the duration of treatment influence microbial community composition. These groupings are key to interpreting ordination and clustering results, allowing differentiation between treatment effects and temporal shifts.

Furthermore, based on Bray–Curtis dissimilarity indexes, a pairwise Bray–Curtis distance matrix was generated showing the dissimilarity between each pair of samples (Table 4).

The Bray–Curtis dissimilarity matrix summarizes the pairwise distances between the eight group samples (BC01 to BC15) included in the present study. Distances range from 0.000 (maximum similarity) to 0.764 (maximum observed dissimilarity). In this matrix, samples BC05 and BC07 exhibit the lowest dissimilarity (0.169), indicating that these two communities are highly similar in composition. In contrast, BC01 and BC13 are the most dissimilar pair (0.764), suggesting a marked shift in community structure between these two samples. Additionally, samples from similar treatments or time points (e.g., BC03 and BC15 with 0.210) often show lower dissimilarity values, hinting at treatment or temporal clustering. These differences may reflect underlying environmental or biological factors differentiating the samples. Overall, the matrix reveals a moderate to high level of compositional variability among the samples.

**Figure 5:**
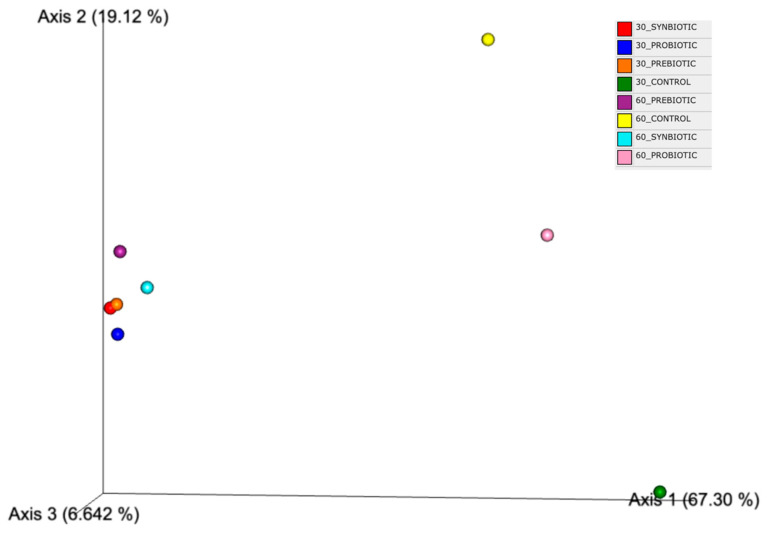
3D Emperor plot of the eight sample groups, based on the Bray–Curtis dissimilarity index at the genus level.

To visually represent the similarities and differences among the microbial profiles of all sample groups, a 3D Emperor plot was generated utilizing the Bray–Curtis dissimilarity index at the genus level. As illustrated in Figure 5, the Bray–Curtis Emperor plot reveals distinct clustering patterns in microbial community composition across different treatment groups and time points. The first control group (baseline) is markedly separated from all other samples, indicating a significant shift in the microbiome over time. Similarly, the second control group (at 60 days) shows a distinct profile compared to both the baseline and the intervention groups, suggesting natural temporal changes in microbial structure. Notably, the probiotic group at 60 days diverges significantly from all other samples, including its earlier 30-day time point. This suggests a delayed but substantial alteration in the microbiome composition specifically driven by the probiotic intervention. In contrast, all other treatment groups—including Synbiotic at 30 and 60 days, Probiotic at 30 days, and Prebiotic at both 30 and 60 days—cluster closely together. This indicates that these interventions induce similar microbial profiles or contribute to the stabilization of the gut microbiota over time. Overall, these findings suggest that while most interventions (Prebiotic, Synbiotic, early Probiotic) exert similar effects on the microbiome, the Probiotic alone at 60 days leads to a distinct microbial shift. Additionally, changes in the Control groups highlight the importance of accounting for natural microbiome fluctuations over time.

**Figure 6:**
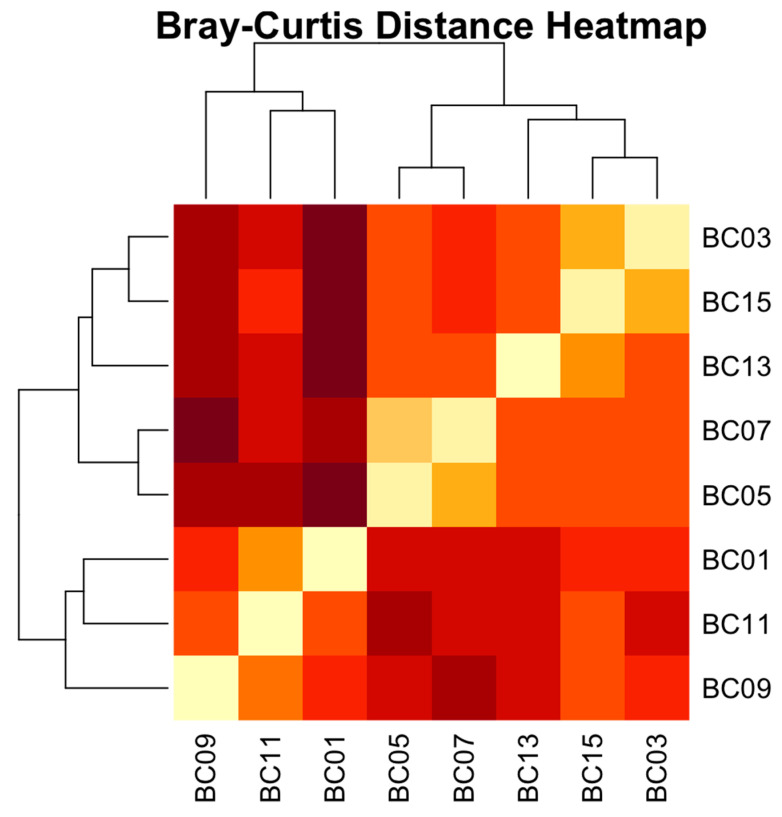
Bray–Curtis dissimilarity heatmap of microbial communities across eight treatment groups after 30 and 60 days. Each cell represents the Bray–Curtis distance between pairs of samples, with darker shades indicating greater dissimilarity.

To further explore microbial community differences among treatment groups, a Bray–Curtis dissimilarity heatmap was generated based on pairwise comparisons of the eight samples. The heatmap visually presents the degree of dissimilarity between each pair of samples, with darker blue shades indicating greater compositional differences and lighter shades representing more similar microbial profiles (Figure 6).

Notably, the samples BC05 and BC07 exhibit the highest degree of similarity, as shown by the bright yellow cells (Bray–Curtis distance = 0.169), suggesting near-identical microbial profiles. Similarly, BC03 and BC15, as well as BC01 and BC11, display low dissimilarities, forming tight sample pairs. These relationships are consistent with the hierarchical clustering results, which group these samples closely on the dendrogram.

In contrast, samples such as BC13 and BC09, or BC05 and BC09, show relatively high dissimilarity values, visualized as the darkest cell. These indicate substantial differences in microbial community structure, possibly reflecting environmental variation, distinct microbial niches, or differential exposure to anthropogenic or ecological factors.

The row and column dendrograms on the heatmap further support the grouping of samples into three main clusters: (1) BC05, BC07, and BC13; (2) BC03 and BC15; and (3) BC01, BC11, and BC09, supporting the notion that both time and treatment type substantially impact microbial community structure.

**Figure 7:**
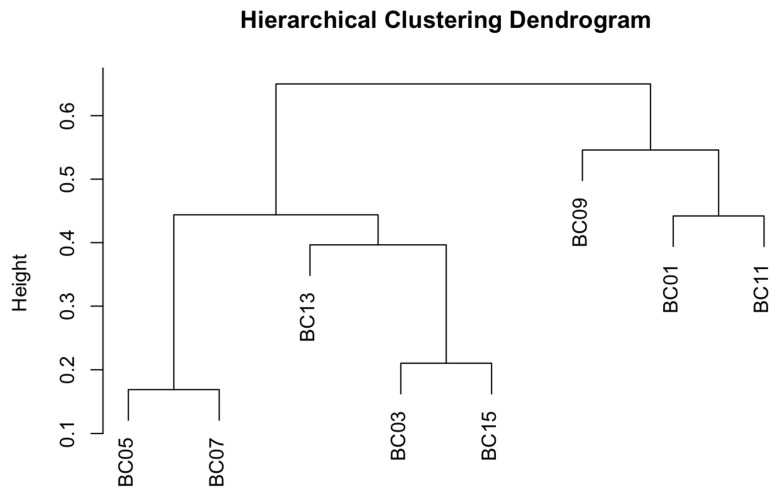
Hierarchical clustering dendrogram of samples based on Bray–Curtis dissimilarities.

Additionally, a dendrogram was generated for the sample groups, allowing for a clearer understanding of relationships across the dataset (Figure 7). Samples are clustered using average linkage (UPGMA), with branch lengths representing relative ecological dissimilarity. The hierarchical clustering dendrogram based on Bray–Curtis dissimilarity illustrates the similarity among samples. The clustering reveals three main groupings: (i) BC05 and BC07, which are the most similar pair; (ii) BC03 and BC15, which cluster closely with BC13; and (iii) BC01 and BC11, which cluster closely with BC09. This suggests that both treatment effects and temporal changes influence community composition.

**Figure 8:**
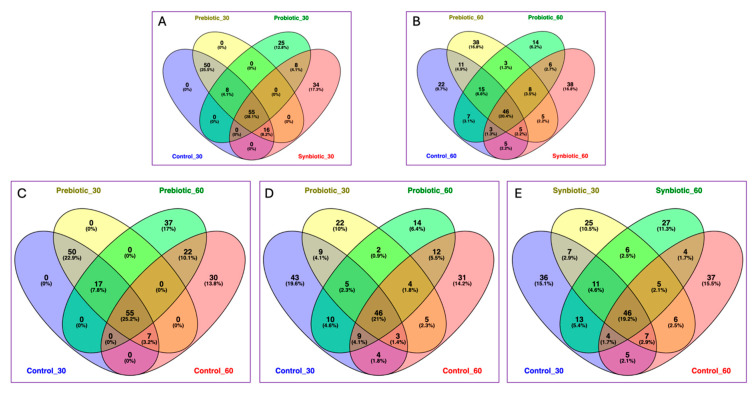
Venn diagrams show shared and unique microbial features among treatment and control groups after 30 and 60 days. (**A**) All treatment groups after 30 days (Control, Probiotic, Prebiotic, Synbiotic). (**B**) All treatment groups after 60 days. (**C**) Prebiotic administration compared across 30 and 60 days with controls. (**D**) Probiotic administration compared across 30 and 60 days with controls. (**E**) Synbiotic administration compared across 30 and 60 days with controls.

Eventually, five Venn diagrams were created using the online tool BioTools.fr (https://www.biotools.fr/misc/venny, accessed on 10 June 2025), which is based only on the composition of bacterial genera and not on their percentage or abundance, allowing the calculation of the intersection in different components. The tool generates a textual output indicating which elements are in each intersection or are unique to a certain list and produces a graphical output in the form of a Venn diagram. Figure 8A illustrates the microbial features shared among all four groups after 30 days: Control_30, Probiotic_30, Prebiotic_30, and Synbiotic_30. A substantial core of 55 features (28.1%) was shared across all groups, indicating some microbiome stability despite treatment. Both Probiotic_30 and Synbiotic_30 had notable unique components (25 and 34 features, respectively), indicating that these treatments induced early microbiome shifts. In contrast, Prebiotic_30 and Control_30 showed no unique features, suggesting high overlap with the other conditions at this time point. However, these two groups share the highest number of unique features (50; 25.5%). After 60 days (Figure 8B), the number of shared features across Control_60, Probiotic_60, Prebiotic_60, and Synbiotic_60 was 46 (20.4%), slightly lower than at 30 days. This suggests a trend toward greater divergence in community structure over time. Prebiotic_60 and Synbiotic_60 showed the highest numbers of unique features (38 each), indicating that these treatments led to pronounced and distinct microbial shifts. Probiotic_60 had a smaller number of unique features (14), suggesting a more moderate or delayed effect. Control_60 also contributed a sizable number of unique features (22), reflecting natural temporal changes in the microbiota. Figure 8C focuses on the prebiotic intervention over time. Prebiotic_60 had a high number of unique features (37; 17%), while Prebiotic_30 had none, indicating that the prebiotic’s impact became more pronounced over time. The shared core of 55 features (25.2%) suggests some baseline consistency across the groups, but Control_60 (30 unique features) also displayed temporal changes. Overall, these results suggest that the prebiotic effect is time-dependent, with a stronger impact evident after 60 days. In this comparison, Probiotic_30 showed more unique features (22; 10%) than Probiotic_60 (14; 6.4%), indicating a stronger or more individualized early response to the probiotic. Control_30 and Control_60 also had substantial unique components (43 and 31, respectively), suggesting natural microbiome variability over time. The shared core across all groups (46 features; 21%) points to some microbial stability, but the data show that probiotic effects are more prominent at 30 days, potentially leveling out by day 60 (Figure 8D). Synbiotic administration resulted in consistent microbial shifts at both time points, with Synbiotic_30 and Synbiotic_60 contributing 25 and 27 unique features, respectively. Control_30 and Control_60 again displayed notable unique features (36 and 37), showing natural changes over time. The shared core (46 features; 19.2%) indicates stable taxa across conditions (Figure 8E). These findings support a sustained and time-progressive impact of synbiotic supplementation on gut microbiota composition.

## 4. Discussion

Considering the significance of the intestinal microbiome in overall well-being, nutritional efficiency, growth, and development of an organism, together with the limited knowledge of the gut microbiota in terrestrial gastropods, the aim of this study is to broaden the understanding of the function of gut microbial communities in the farmed snail *Cornu aspersum maxima*. In the present study, we examine the effects of dietary patterns incorporating the probiotic *L. plantarum* and the prebiotic inulin, aiming to assess the diversity and anticipated functionality of the bacterial communities in the gut of the farmed snail *Cornu aspersum maxima* under different feeding regimes. Additionally, we compared the effects of these dietary factors on the growth of the snails while attempting to identify potential pathogenic bacteria that could affect them.

It follows from this study that all groups showed a slight weight gain up to day 30, followed by a decrease by day 60. The gut microbiome of *Cornu aspersum maxima* is highly diverse and responsive to dietary interventions, primarily composed of the phyla Proteobacteria and Actinobacteria and in less abundance, Firmicutes and Bacteroidota. Comparison with other gastropods highlights both common and divergent microbial features. In the freshwater invasive snail *Pomacea canaliculata*, it reported that the gut microbiota was dominated by Proteobacteria (51.6%) and Bacteroidetes (23.6%), with Ochrobactrum and Sediminibacterium being the most abundant genera [2]. In our terrestrial model (*Cornu aspersum maxima*), the gut community was likewise rich in Proteobacteria but also showed a remarkable increase in Actinobacteria and several unclassified taxa. These differences likely reflect ecological (aquatic vs. terrestrial) and dietary adaptations, suggesting both functional convergence and species-specific divergence in snail gut microbiomes. Supplementation with *L. plantarum* altered the microbial composition, notably increasing beneficial taxa such as Actinobacteria while reducing potentially pathogenic Proteobacteria. In contrast, feeding with inulin and synbiotics demonstrated reduced abundances of both the main phyla Proteobacteria and Actinobacteria. Although synbiotic treatment enhanced microbial diversity (unassigned 22.98%), it was also associated with the highest mortality rate, suggesting that greater diversity does not necessarily correlate with improved health outcomes in terrestrial snails. Interestingly, *L. plantarum* was not detected post-administration, implying limited colonization or transient activity. Inulin promoted the growth of unclassified but potentially beneficial genera, underscoring its role in gut microbiome restructuring. Although the detection of genera often associated with pathogenicity such as *Klebsiella* and *Mycoplasma* raises concerns about snails as potential vectors of zoonotic agents, their abundance decreases significantly after the administration of additives.

In assorted studies, several feed formulations with different ingredient ratios have been administered to snails. Garcia et al. [22] concluded that artificial diets are more suitable for snail growth compared to the simple provision of fresh vegetable leaves, as they contain higher levels of protein and calcium carbonate. In earlier experiments, Sampelayo et al. [52] found that when diets with a protein content greater than 17% were administered to *Cornu aspersum* snails, food intake by snails decreased without an increase in food conversion efficiency or snail growth. In our own experiment, the low rate of mass change is therefore expected, as it aligns with the results of previous studies, as well as with more recent research conducted on the same species providing the same diet [53].

The initial slight increase in the weight of snails up to 30 days, followed by a decrease from 30 to 60 days in all snail groups, can be attributed to several factors related to diet, environmental conditions, and the physiology of the animals. During the early days of the experiment, rapid growth may be observed due to their successful adaptation to the new diet. Additives such as inulin and *L. plantarum* may enhance the digestive process and nutrient absorption in the initial phase; however, this improvement may not be permanent, as the animals adapt both to the new diet and to their gut microbiome. Fiber-rich diets tend to reduce food intake and increase the viscosity of the digestive system while decreasing digestibility [54]. Gidenne [55] also stated that the primary role of dietary fiber fractions in animal nutrition lies in their effect on the rate of passage, mucosal function, and their role as a substrate for the gut microbiota, which is linked to performance and digestive health. Elevated fiber levels in the diet make it less desirable for non-ruminant animals such as pigs, poultry, and snails.

Prebiotics and probiotics can support the growth and proliferation of “beneficial” bacteria in the gut, enhancing the digestive process and nutrient absorption during the initial phase of the study, leading to an increase in body weight. However, continued administration of these additives may lead to saturation or modifications in the gut microbiota, which could result in reduced nutrient absorption such as calcium and phosphorus [56]. This, in turn, may limit the ability of the snails to continue increasing their body weight. Currently, there is limited or no data available concerning the fiber nutrient requirements of snails, as is the case for other conventional livestock species.

Snails may transition from a phase of rapid weight gain (due to initial adequate food intake) to a phase of more stable growth or a normal slowing of development, where weight gain decreases or stops. This may occur due to the maturation process or a slowing of their metabolism as the animals adapt to their daily routines [57,58]. Furthermore, they may have depleted the energy reserves accumulated during the initial phase of growth and may no longer be able to replenish these reserves due to reduced nutrient absorption [59,60]. Moreover, although the experiment is carried out under controlled environmental conditions, the snails may undergo environmental stress, which may not instantly appear but could become noticeable after 30 days, once the initial adjustment phase is complete and the adverse effects of this stress become more evident [61,62].

An interesting outcome was observed after the administration of *L. plantarum*, as the Probiotic group had a significantly lower mortality rate in comparison to the Control group, which can be attributed to the protective effects of *L. plantarum*. The mortality of snails after being fed with *L. plantarum* and inulin could be attributed to a range of factors, such as overdose, changes in the gut microbiome, undesirable interactions with other foods or substances, or even immune responses. In this specific experiment, the overdose scenario is not applicable, as doses were based on both our previous studies [48] and those of other researchers using the same diet in the same species [25,36,49]. Additionally, it is important to note the stability of environmental factors (humidity, temperature, cleanliness of the environment) to which the snails were subjected, as well as their ad libitum access to food. Certain animals, including snails, may react negatively to specific bacteria or microorganisms; however, *L. plantarum* and various strains of the genus are not recognized as pathogenic or foreign bodies that could induce an excessive immune response, leading to inflammation or even mortality [25,36]. Additionally, the stress factor can also affect the mortality rate and be a possible reason for the observed results in combination with observed differences in the microbiome composition.

The comparison of the microbiome between the Control groups revealed apparent differences in Bray–Curtis distances in the composition and abundance of bacterial populations, although no drastic changes were observed. The dominant phylum across all terrestrial gastropods was Proteobacteria. The minor fluctuations in their abundance over time suggest the natural dynamics of the microbiome. Firmicutes maintained a stable presence, while Actinobacteria displayed variation, which may be attributed to random fluctuations or adaptive changes within the microbiome over time [63,64]. Bacteroidota remained at consistently low levels with no significant changes.

Upon comparing the other groups to the Control group, it was observed that prebiotics and synbiotics appear to improve microbial balance by reducing potentially pathogenic groups and enhancing beneficial microbes, mainly after 60 days of treatment. These differences suggest that the interventions had a more pronounced effect on the microbiome than the natural fluctuations observed in the Control group. The microbiome analysis of the 30_CONTROL and 60_CONTROL groups at the genus level revealed distinct changes in bacterial composition, with certain genera maintaining their presence, while others showed variations in abundance. In the 30_CONTROL group, *Cutibacterium* was the most abundant genus, recognized for its synbiotic relationship with human skin and its production of propionic acid [65]. This was followed by *Lawsonella*, *Staphylococcus*, *Enterobacter*, and *Klebsiella*, all of which are typically associated with the gut microbiome and may have pathogenic potential [66,67]. Additionally, genera such as *Streptococcus*, *Corynebacterium*, and *Lactococcus* were identified, playing key roles in fermentation and the maintenance of microbial balance [68,69].

In the 60_CONTROL group, alterations in bacterial composition were observed, including an increase in the abundance of *Buttiauxella*, as well as a notable presence of *Enterobacter* and *Cutibacterium*, although the latter showed a substantial decrease. Additionally, there was a slight rise in unclassified (unassigned) bacteria—bacterial groups either insufficiently represented in the *SILVA* database or belonging to rare and less accessible species. The observed decrease in *Actinobacteria* and the increase in unclassified bacteria may be attributed to several factors, including (a) changes in the gut environment resulting from dietary differences or altered intestinal motility [70]; (b) a reduction in the competitive advantage of certain bacterial species, allowing for the proliferation of rarer or more adaptive species [71]; and (c) a potential decline in bacteria that require specific environmental conditions, such as low oxidative stress or specialized nutrients, for survival [72].

Additionally, new genera such as *Allorhizobium*, *Neorhizobium*, *Pararhizobium*, and *Rhizobium*, which engage in nitrogen fixation processes, were detected [73,74]. The presence of *Sphingobacterium*, known for its role in lipid and polysaccharide metabolism, was also recorded [75,76]. Notably, *Acinetobacter* species [77,78], which contribute to cellulose degradation, as well as *Pseudomonas* species, involved in the breakdown of polysaccharides and organic compounds [79,80], were observed. Finally, *Brevundimonas* was identified, which is known for its ability to degrade pharmaceutical waste and pesticides [81,82]. These findings illustrate the dynamic nature of the microbiome in the Control groups and suggest ecological shifts in bacterial communities over time, potentially driven by environmental and metabolic changes.

In the groups that received prebiotic, significant changes in the microbiome composition were observed, which may be attributed to the effects of inulin. While Proteobacteria remained one of the dominant bacterial phyla, a tendency for their reduction was noted at 60 days, suggesting a potential improvement in microbial balance, as certain species within this phylum are associated with potentially pathogenic microorganisms. Firmicutes aligning with the enhancement of bacteria involved in carbohydrate fermentation and the production of short-chain fatty acids showed a slight decrease [83]. An unexpected shift was observed in the abundance of Actinobacteria, which, contrary to expectations of an increase due to the presence of beneficial genera such as *Bifidobacteria*—known to thrive in the presence of prebiotics—showed a slight decrease [49]. This finding may suggest the presence of competing microbial pressures or an insufficient effect of the prebiotic intervention at this stage. In contrast, Bacteroidota maintained a higher abundance showing significant fluctuations. Overall, the results suggest that prebiotic supplementation may positively impact microbiomes by promoting bacteria associated with gut health.

In the 30_PREBIOTIC group, the most abundant genera included *Klebsiella*, *Enterobacter*, and *Cutibacterium*, which are associated with infections, polysaccharide fermentation, and propionic acid production, respectively. In a previous study that examined the effects of prebiotic supplementation on the feline gut microbiota, it was observed that prebiotic inclusion led to an increase in beneficial bacteria, such as *Bifidobacterium* and *Lactobacillus*. However, it also noted a slight increase in *Enterobacteriaceae*, including *Klebsiella*, which highlights the complex effects of prebiotics on gut microbiota composition [84]. *Lactococcus*, a genus involved in lactic acid fermentation, was also present, as well as *Buttiauxella*, *Escherichia*, and *Shigella*, which play roles in gut homeostasis regulation or may contribute to disease under certain conditions. Recent studies have highlighted the significant impact of prebiotic supplementation on the gut microbiota composition in various animal species, including birds. In a 2021 study analyzing the fecal microbiota of six bird species, researchers identified several genera with notable roles. The study also detected potential pathogenic bacteria such as *Burkholderia*, *Escherichia*, *Shigella*, and *Ochrobactrum*. While these bacteria are part of the normal gut microbiota, their overgrowth or presence in certain contexts can be associated with disease [85]. In the 60_PREBIOTIC group, while *Klebsiella* and *Enterobacter* remained dominant, a significant difference was observed with the appearance of an unclassified genus (unassigned). Additionally, new genera such as *Dysgonomonas* and *Macellibacteroides*, which engage in lipid and protein processing primarily through anaerobic fermentation, were detected [86,87]. Furthermore, *Shewanella*, which is typically found in marine gastropods, was identified [88]. These findings suggest that certain bacterial species may function as common symbionts across different snail species, pointing to a potentially shared microbial community among these organisms. These observations indicate a dynamic reshaping of the bacterial community, driven by prolonged exposure to prebiotics, which fosters the growth of specific microorganisms and impacts the metabolic activity of the microbiome.

In the probiotic groups, Actinobacteria, which includes beneficial bacteria such as Bifidobacteria, showed a significant increase at 60 days, indicating successful colonization and enhancement of probiotic microorganisms. Simultaneously, Firmicutes exhibited a slight decrease in abundance, supporting the idea that probiotics contribute to maintaining a balanced microbiome. Moreover, Proteobacteria displayed a decreasing trend, which may reflect the regulation of potentially pathogenic species, a favorable outcome for gut health [89,90]. Bacteroidota remained at low levels, with no substantial fluctuations. Overall, the findings suggest that probiotic supplementation enhances the presence of beneficial bacteria, contributing to the establishment of a healthier microbiome at 60 days compared to 30 days.

In the 30_PROBIOTIC group, the predominant genera included *Klebsiella* and *Enterobacter*, which are members of the Enterobacteriaceae family and are typically associated with the intestinal environment and the metabolic activities of probiotic bacteria. High abundance was also observed for the unclassified genus (unassigned), as well as *Cutibacterium* and *Staphylococcus*, bacteria involved in both skin and intestinal processes [91]. In the 60_PROBIOTIC group, significant shifts in the bacterial composition were observed, with *Cutibacterium* becoming the dominant genus, while the unclassified genus remained elevated. *Enterobacter* and *Klebsiella* showed a noticeable decrease, whereas *Buttiauxella*, associated with the degradation of organic compounds [92], increased in abundance. Additionally, new genera such as *Micrococcus*, *Acinetobacter*, and *Arcticibacter* became more prominent, suggesting an adaptation of the microbiome to prolonged probiotic administration. While direct studies on the effects of prolonged probiotic administration specifically increasing the abundance of these genera in non-human animals are limited, related research offers insights into the impact of probiotics on gut microbiota composition [93]. These changes highlight a dynamic reshaping of the microbial community over time, driven by the effects of probiotics in regulating bacterial balance.

The comparison between the 30_SYNBIOTIC and 60_SYNBIOTIC groups reveals significant shifts in bacterial composition, influenced both by the administration of synbiotics and the passage of time. Actinobacteria, which includes beneficial bacteria such as Bifidobacteria, showed a marked decrease at 60 days, contradicting our hypothesis that the combination of prebiotics and probiotics promotes the colonization and proliferation of beneficial microorganisms. Firmicutes exhibited a slight increase in abundance, which could be associated with enhanced carbohydrate fermentation and the production of short-chain fatty acids, thus contributing to improved gut health [94]. Conversely, Proteobacteria showed a decreasing trend at 60 days, reflecting the regulation of potentially pathogenic microorganisms by the synbiotic intervention. Bacteroidota remained at low levels, with no significant changes observed.

In the 30_SYNBIOTIC group, the most abundant genera included *Klebsiella* and *Enterobacter*, both of which belong to the Enterobacteriaceae family and are linked to the intestinal microbiome. Additionally, a notable presence of *Cutibacterium*, as well as *Methylobacterium* and *Methylorubrum*—bacteria involved in methanol metabolism—was recorded [95,96]. Furthermore, genera such as *Lactococcus* and *Microbacterium*, which participate in fermentation processes and contribute to the protective functions of the microbiome, were also present [97]. Additionally, *Curtobacterium* is a genus of bacteria comprising both pathogenic and beneficial species. Recent studies have provided insights into their genomic characteristics, pathogenicity, and potential applications [98,99,100]. In the 60_SYNBIOTIC group, although *Klebsiella* and *Enterobacter* remained dominant, their abundances decreased, while the proportion of unclassified bacteria (unassigned) increased. Additionally, *Buttiauxella*, a genus involved in the breakdown of organic compounds [92,101], was detected, along with *Modestobacter*, which is associated with resilient microbial ecosystems in harsh environmental conditions [102,103], and *Mycoplasma,* which is a genus primarily associated with pathogenicity in humans and animals [104]. Notably, the presence of *Lelliottia*, a member of the *Enterobacteriaceae* family and a primary pathogen in plants [105], as well as *Staphylococcus*, which is part of the human microbiota, was observed. These changes suggest that prolonged synbiotic administration leads to an adaptive reshaping of the microbiome, reflecting a dynamic reorganization of the microbial community over time. Table 5 lists the most abundant genera and their primary functional role in the *Cornu aspersum maxima* species.

Although synbiotics are generally regarded as beneficial, several studies have documented unexpected or even adverse outcomes. In BTBR mice, synbiotic consumption increased intestinal permeability and altered behavioral and microbial parameters, indicating detrimental physiological consequences [106]. In broiler chickens, in ovo administration of a commercial synbiotic reduced hatchability, presumably due to nutritional competition or toxic metabolic byproducts [107]. Likewise, in a rat model of dextran sulfate sodium-induced colitis, a synbiotic mixture of *Bacillus licheniformis* and *Saccharomyces cerevisiae* extract aggravated intestinal inflammation instead of alleviating it [108]. These findings underline that synbiotic effects can be highly host- and context-dependent, reinforcing the importance of cautious evaluation before their application in non-model invertebrates such as snails.

In our study, the probiotic administered to the snails was *Lactobacillus plantarum*. Although there is evidence that some *Lactobacillus* species, including *L. plantarum*, may be present in the microbiome of the digestive system, although they are not always dominant, it was experimentally added as a dietary supplement. *L. plantarum* has been used in previous studies involving the land snail *Cornu aspersum maxima*; however, the primary objective of its administration was to evaluate its probiotic properties and its impact on the snail microbiota, rather than to precisely characterize the microbiome itself. According to the existing literature, only a fraction of the strains isolated from the snail’s digestive tract exhibited functional attributes indicative of potential probiotic activity [25]. Furthermore, the supplementation of *L. plantarum* (Sgs14 strain) in snails may contribute to improved gut health by reducing pathogenic bacteria, enhancing the digestion of polysaccharides and dietary fibers, and boosting the immune system through the production of antimicrobial peptides [36].

Despite the potential benefits, sequencing did not detect the presence of *L. plantarum* in any of the experimental groups, raising questions about the bacterium’s ability to establish a permanent presence in the snail microbiome. The absence of this bacterium from the samples may indicate its expulsion from the organism after a certain period. The strains present in the administered formulation may have lacked the necessary adhesion properties to the intestinal mucosa, which are critical for their probiotic efficacy. This fact may be associated with the increased mortality observed with symbiotic administration. In human studies, the fate of ingested *L. plantarum* has been examined, revealing its presence in feces, which may suggest expulsion after passage through the gastrointestinal tract [109]. The inability of *L. plantarum* to establish permanent colonization in the snails’ gut suggests that it may only be effective during the period of administration, without maintaining a stable presence in the digestive system, as is often observed with many probiotic bacteria such as *Lactobacillus plantarum* Sgs14, which has been demonstrated to exhibit high adhesion rates (>70%), making it a highly suitable candidate probiotic strain [25]. Furthermore, in snail farming environments, the presence of pathogenic bacteria such as *Pantoea* and *Klebsiella* has been associated with a reduction in the *L. plantarum* concentration, suggesting a potential competitive interaction between the microorganisms [110].

The methods employed in this study focused on the identification of dominant bacteria through metagenomic analysis. Therefore, if *L. plantarum* was present in exceptionally low quantities, it may not have been detected due to the limitations of the methodology. Recent and previous literature highlights the possibility that some invertebrates, including gastropods, may not sustain a stable or resident gut microbiome at all. In aquatic invertebrates, gut microbial communities are often composed of transient or ingested organisms, rather than established symbionts [111]. In the terrestrial snail *Pomacea canaliculata*, compartmentalized gut segments such as the buccal mass, stomach, and intestine host distinct and relatively low-diversity microbial populations, lacking a consistent core microbiome [2]. A comprehensive review of microbial diversity in snails further underlines the scarcity of well-defined and persistently present microbial taxa in these organisms, pointing to the potential absence of a stable microbiome [112]. Other molluscan studies support this notion, indicating that host–microbiome interactions in many gastropods are likely environment-dependent and lack long-term stability [113]. Consequently, there appears to be a significant research gap regarding the precise interaction between *L. plantarum* and snails, as well as its relationship with their native microbiota.

Thus, it is evident that further investigation is necessary to ascertain whether *L. plantarum* can establish a permanent colonization within the digestive system of snails or if it functions exclusively as a temporary probiotic during supplementation. Gaining a deeper understanding of this interaction could provide crucial insights for the use of probiotics in promoting the health and breeding of snails.

**Table 5:** Dominant bacterial genera and their main functional role.

Genus	Functional Role
*Pseudomonas*	Present in all terrestrial snails. Contributes to cellulolytic activity and the degradation of plant fibers [114].
*Acinetobacter*	Involved in cellulose degradation [78].
*Brevundimonas*	Degradation of pharmaceutical waste and insecticides [115].
*Cutibacterium*	Synbiotic with human skin, produces propionic acid [116,117].
*Lawsonella*	Emerging human pathogen, associated with infections, especially following medical procedures such as autologous fat grafting [118].
*Staphylococcus*	Protection against other pathogens [119].
*Enterobacter*	Fermentation of polysaccharides [120].
*Klebsiella*	Associated with infections and reduced presence of beneficial *Lactobacillus* bacteria [110].
*Streptococcus*	pH regulation via lactic acid production [121].
*Corynebacterium*	Degradation of organic matter. Protection from pathogens [122].
*Lactococcus*	Fermentation of plant materials and sugars via lactic acid production [123,124].
*Allorhizobium*, *Neorhizobium*, *Pararhizobium*, *Rhizobium*	Nitrogen fixation [125].
*Buttiauxella*	Exclusively in land snails. Associated with pathogenic conditions [112].
*Escherichia*	May include pathogenic strains [126].
*Shigella*	Pathogens cause intestinal infections [127].
*Dysgonomonas*	Digestion of plant fibers [128].
*Macellibacteroides*	Digestion of plant materials through anaerobic fermentation [129].
*Shewanella*	Primarily in marine snails. Involved in the breakdown of organic matter [130].
*Micrococcus*	Degradation of organic matter. Protection from pathogens [131].
*Arcticibacter*	Survives in low temperatures. Degrades fats [132].
*Methylobacterium, Methylorubrum*	Degradation of methanol [133].
*Microbacterium*	Degradation of organic compounds [134].
*Curtobacterium*	Degradation of plant fibers. Potentially pathogenic [135,136].
*Modestobacter*	Recycling of organic matter and degradation of organic compounds in soil and plants [137].
*Mycoplasma*	Causes diseases in the respiratory, reproductive, and urinary systems [138].
*Lelliottia*	Causes infections primarily in plants [105].
*Sphingobacterium*	Metabolism of lipids and polysaccharides [75,139].

## 5. Conclusions

This study demonstrates that the intestinal microbiome of *Cornu aspersum maxima* is highly responsive to dietary interventions with probiotics, prebiotics, and synbiotics. Supplementation with *Lactobacillus plantarum* increased the relative abundance of Actinobacteria and reduced potentially pathogenic Proteobacteria, suggesting a protective modulation of the gut ecosystem. Inulin, on the other hand, promoted the emergence of unclassified and novel genera, indicating a restructuring of microbial communities that may reflect a broader ecological impact on the snail gut.

Despite these microbial changes, improvements in growth were limited across all treatments, and weight gain declined after 30 days. Importantly, the synbiotic treatment, while enhancing microbial diversity, was consistently associated with the highest mortality, pointing to adverse interactions when probiotics and prebiotics were combined under the tested conditions.

Overall, these results indicate that targeted dietary supplementation can shape the gut microbiota of terrestrial snails and potentially reduce pathogenic pressures. However, the lack of positive effects on growth and the negative outcome of synbiotic supplementation underline the need for cautious evaluation before such strategies are applied in snail farming. Future work should explore strain-specific properties, optimal dosages, and long-term effects to design safe and effective microbiome-based interventions that improve both productivity and survival.

In the present study, a comprehensive analysis of the gut microbiome of the farmed snail *C. aspersum maxima* was conducted. The results can be summarized in some significant key points:

- The intestinal microbiome of *C. aspersum maxima* is particularly diverse and sensitive to dietary interventions. It is primarily composed of the bacterial phyla Proteobacteria (formerly Proteobacteria) and Actinobacteria, with Firmicutes and Bacteroidota present in lower abundances.
- Significant differences in microbial composition were observed between groups and time points, as shown by Bray–Curtis, NMDS, and Venn analyses.
- The presence of unassigned microorganisms increased in all supplemented groups, especially in the inulin group (Prebiotic), reaching 35%.
- *L. plantarum* was not detected in any of the groups after administration, indicating a transient presence or the inability to colonize the snail gut.
- Only the administration of *L. plantarum* increased the presence of the phylum Actinobacteria, which includes beneficial bacteria, and decreased the presence of potentially pathogenic Proteobacteria.
- Administration of inulin (Prebiotic) caused an increase in unclassified genera and the appearance of new species (e.g., *Dysgonomonas*, *Macellibacteroides*, *Shewanella*), indicating a restructuring of the microflora.
- Co-administration of a probiotic and a prebiotic (synbiotic) significantly enhanced microbial diversity but also resulted in a dramatic increase in mortality after 60 days.
- Weight gain was limited and decreased after the initial phase (30 days), possibly due to adaptation or saturation of the microflora.
- The weight gain rate (WGR) was highest in the Control group, while it was lower in the Probiotic- and Prebiotic-supplemented groups, particularly after 60 days.
- Synbiotics may induce adverse synergistic effects, possibly due to the inability of the probiotic to establish stable colonization and the resulting competition within the microbiome.
- The abundance of potentially pathogenic genera was significantly reduced in the groups receiving probiotics or prebiotics, suggesting a potential protective effect of the dietary supplements on the microbiome.
- Beta diversity analyses confirmed that microbial communities clustered by treatment and time, indicating strong effects of diet and exposure duration on the structure of the gut microbiome.
- Further research is required to better understand the effects of probiotics and prebiotics on snail biology and to develop safe dietary interventions.
- The use of synbiotics in snails should be reviewed in detail, as their combined administration may be detrimental to survival.

## Figures and Tables

**Table 1:** Composition and nutrient levels of the ration used.

Composition					
Corn, Soybean Flour *, Calcium Carbonate, Monocalcium Phosphate, Sodium Chloride, Sodium Bicarbonate					
Detailed Ingredients (in %)					
Total nitrogenous substances	20.00%		Calcium (Ca)	1.70%	
Total fats	3.80%		Phosphorus (P)	0.85%	
Total fibrous substances	5.00%		Sodium (Na)	0.20%	
Total ash	8.70%		Lysine	1.10%	
Humidity	13.00%		Methionine	0.50%	
Additives (per Kg of forage)					
Vitamins			Trace Elements		
3a672a Vitamin A	I.U.	9.70	3b603 Ζinc oxide [ZnO]	mg	90
3a671 Vitamin D	I.U.	3.0	3b606 Hydrated zinc amine chelate complex	mg	27
3a700 Vitamin E	mg	60	3b202 Calcium iodate	mg	1.13
3a711 Vitamin K_3_ (MNB)	mg	3.0	3b801 Sodium selenite	mg	0.38
3a821 Vitamin B_1_	mg	1.1	3b103 Iron sulfate	mg	150
Vitamin B2 (Riboflavin)	mg	4.0	3b106 Iron from hydrated amino acid chelate complex	mg	44
3a831 Vitamin B_6_	mg	4.50	3b502 Μanganese oxide	mg	52.50
Vitamin B_12_ (Cyanocobalamin)	mg	0.02	3b506 Μanganese from glycine chelate	mg	34.50
3a314 Niacin (Νicotinic acid)	mg	22.50	3b404 Copper oxide	mg	2.50
3a841 Calcium pantothenate	mg	15			
3a316 Folic acid	mg	0.38			
3a880 Biotin	mg	0.08			
3a890 Cholinechloride	mg	300			
Digestibility enhancer	Fytase EC 3.1.3.26				

* Soybean flour comes from genetically modified soybeans, the import and processing of which is allowed freely in the European Union.

**Table 2:** Growth performance (weight, weight gain rate) and mortality of *Cornu aspersum maxima* at 30 and 60 days under different dietary treatments.

Trait	Control	Probiotic	Prebiotic	Synbiotic
Initial weight (g)	9.87 ± 1.20	9.44 ± 1.33	10.37 ± 1.29	10.71 ± 1.95
30-Day weight (g)	11.27 ± 1.64	10.49 ± 1.64 ^a^	11.81 ± 1.65	12.13 ± 2.28
60-Day weight (g)	10.76 ± 1.51	9.72 ± 1.41	10.54 ± 1.59	11.54 ± 1.84
30-Day weight growth rate—WGR (%)	14.18 ± 16.67	11.13 ± 17.45	13.89 ± 15.99	13.27± 21.43
60-Day weight growth rate—WGR (%)	8.99 ± 15.34	2.97 ± 14.94	1.58 ± 15.33	7.76 ± 17.24
30-Day mortality (%)	5.71 ± 1.90	14.29 ± 4.59 ^a^	20 ± 5.58 ^b^	48.57 ± 11.79 ^c^
60-Day mortality (%)	68.57 ± 16.01	51.43 ± 10.33 ^a^	62.86 ± 11.53	100 ± 14.34 ^c^

Values are means ± SD. a, b, c indicate *p* < 0.05 compared to Control.

**Table 3:** Barcodes corresponding to treatment group for each of the eight samples analyzed.

Barcodes	Treatment Group
BC01	30_CONTROL
BC03	30_PROBIOTIC
BC05	30_PREBIOTIC
BC07	30_SYNBIOTIC
BC09	60_CONTROL
BC11	60_PROBIOTIC
BC13	60_PREBIOTIC
BC15	60_SYNBIOTIC

**Table 4:** Pairwise Bray–Curtis dissimilarity matrix between samples BC01 to BC15.

	BC01	BC03	BC05	BC07	BC09	BC11	BC13	BC15
BC01	0.000	0.680	0.732	0.711	0.614	0.442	0.764	0.673
BC03	0.680	0.000	0.414	0.457	0.606	0.572	0.439	0.210
BC05	0.732	0.414	0.000	0.169	0.698	0.646	0.457	0.413
BC07	0.711	0.457	0.169	0.000	0.752	0.617	0.479	0.443
BC09	0.614	0.606	0.698	0.752	0.000	0.478	0.700	0.575
BC11	0.442	0.572	0.646	0.617	0.478	0.000	0.575	0.445
BC13	0.764	0.439	0.457	0.479	0.700	0.575	0.000	0.354
BC15	0.673	0.210	0.413	0.443	0.575	0.445	0.354	0.000

Notes: The values represent the ecological dissimilarity between each pair of samples based on their community composition, where 0 indicates identical composition and values closer to 1 indicate greater dissimilarity.

## Data Availability

The original data presented in the study are openly available in FigShare at 10.6084/m9.figshare.30011071.
